# A semi-synthetic neolignan derivative from dihydrodieugenol B selectively affects the bioenergetic system of *Leishmania infantum* and inhibits cell division

**DOI:** 10.1038/s41598-019-42273-z

**Published:** 2019-04-16

**Authors:** Maiara Amaral, Fernanda S. de Sousa, Thais A. Costa Silva, Andrés Jimenez G. Junior, Noemi N. Taniwaki, Deidre M. Johns, João Henrique G. Lago, Edward A. Anderson, Andre G. Tempone

**Affiliations:** 1 Centre for Parasitology and Mycology, Instituto Adolfo Lutz, São Paulo, 01246-000 Brazil; 2 Institute of Environmental, Chemical and Pharmaceutical Sciences, Federal University of São Paulo, São Paulo, 09972-270 Brazil; 3 Centre of Natural and Human Sciences, Federal University of ABC, Santo André, 09210-580 Brazil; 4 Hospital das Clínicas HCFMUSP, Faculdade de Medicina, Universidade de Sao Paulo, São Paulo, 05403-000 Brazil; 5 Laboratory of Electron Microscopy, Instituto Adolfo Lutz, São Paulo, 01246-000 Brazil; 6 Department of Biomedical Sciences, Oregon State University, Corvallis, Oregon, 97331 USA; 7 Chemistry Research Laboratory, University of Oxford, 12 Mansfield Road, Oxford, OX1 3TA UK

## Abstract

Leishmaniasis is a neglected disease that affects more than 12 million people, with a limited therapy. Plant-derived natural products represent a useful source of anti-protozoan prototypes. In this work, four derivatives were prepared from neolignans isolated from the Brazilian plant *Nectandra leucantha*, and their effects against intracellular amastigotes of *Leishmania* (*L*.) *infantum* evaluated *in vitro*. IC_50_ values between 6 and 35 µM were observed and *in silico* predictions suggested good oral bioavailability, no PAINS similarities, and ADMET risks typical of lipophilic compounds. The most selective (SI > 32) compound was chosen for lethal action and immunomodulatory studies. This compound caused a transient depolarization of the plasma membrane potential and induced an imbalance of intracellular Ca^2+^, possibly resulting in a mitochondrial impairment and leading to a strong depolarization of the membrane potential and decrease of ATP levels. The derivative also interfered with the cell cycle of *Leishmania*, inducing a programmed cell death-like mechanism and affecting DNA replication. Further immunomodulatory studies demonstrated that the compound eliminates amastigotes via an independent activation of the host cell, with decrease levels of IL-10, TNF and MCP-1. Additionally, this derivative caused no hemolytic effects in murine erythrocytes and could be considered promising for future lead studies.

## Introduction

Leishmaniasis, a neglected tropical disease caused by protozoan parasites of the *Leishmania* genus, affects more than 12 million people worldwide. Currently, this disease is present in 98 countries and it is estimated that 60,000 new cases occur every year in Latin America alone^1^. Human visceral leishmaniasis (VL) is the most severe clinical form of the disease, affecting internal organs such as the spleen, liver, bone marrow and lymph nodes. Usually, VL is fatal within two years without treatment, and the number of deaths ranges from 20,000 to 50,000 people annually^1,2^. The VL chemotherapeutic arsenal comprises just three main drugs (pentavalent antimonials, amphotericin B and miltefosine), which exhibit several limitations, including long administration regimens, hospitalization, high costs and severe adverse effects^3^. The search for new drugs therefore remains a necessity, especially for developing countries.

**Figure 1:**
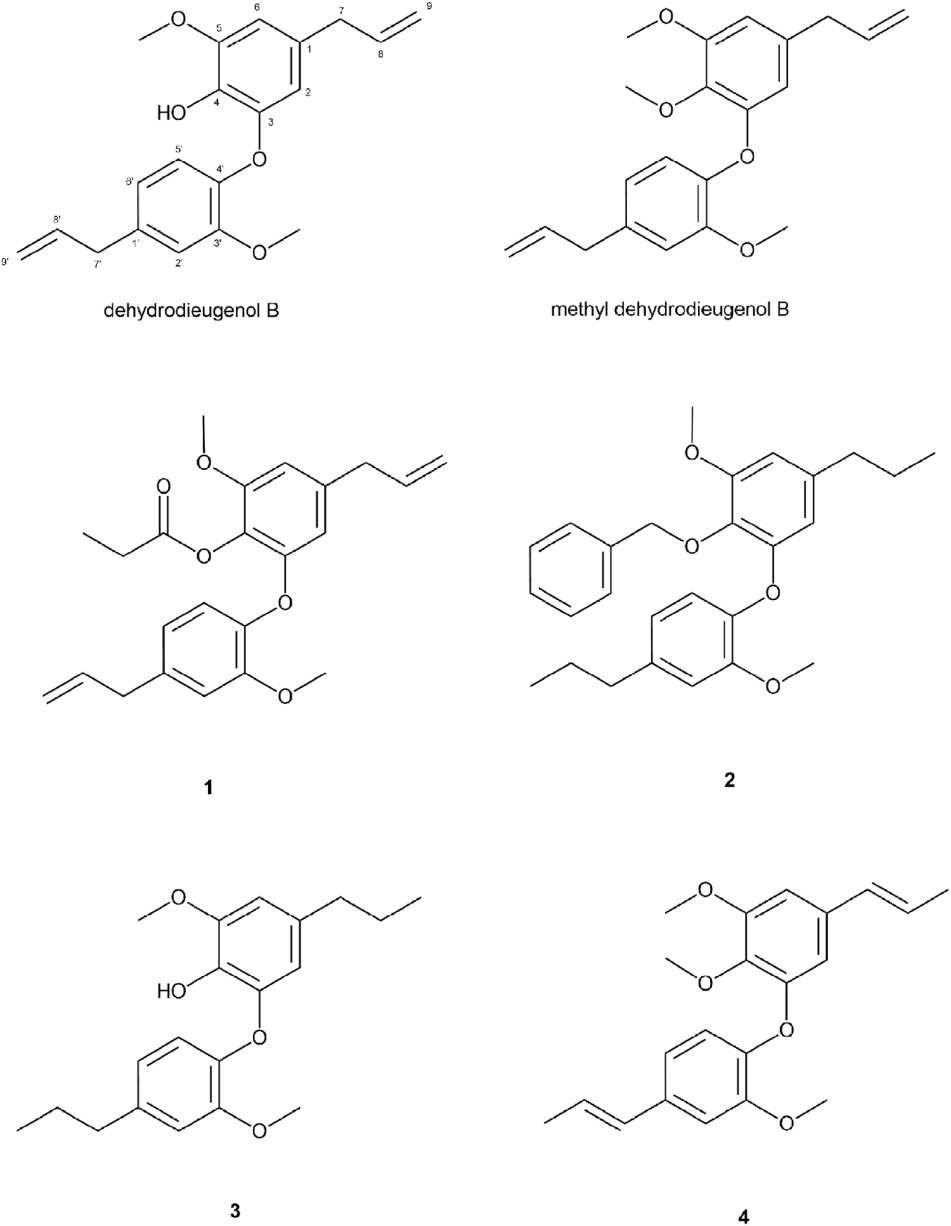
Chemical structures of natural products dehydrodieugenol B, methyl dehydrodieugenol B and semi-synthetic compounds **1**–**4**.

In this context, natural products are excellent prototypes for the synthesis of potent antiparasitic derivatives^3,4^, and indeed around 50% of all FDA-approved drugs are based in some form on natural product scaffolds^5^. In the search for such natural leads against VL, we previously described the anti-*L*. (*L*.) *donovani* activity of neolignans isolated from *Nectandra leucantha* Nees & Mart^6^. Among these, dehydrodieugenol B (Fig. 1) was the most selective, and also presented a promising immunomodulatory effect for visceral leishmaniasis. Recently, we described the anti-*Trypanosoma cruzi* activity of dehydrodieugenol B and its natural methylated derivative, and an *in silico* analysis revealed a promising safety profile for these compounds^7,8^. In continuation of this work, we here report the preparation and biological evaluation of four new semi-synthetic derivatives (**1**–**4**) of dehydrodieugenol B and methyl dehydrodieugenol B. The anti-*L*. (*L*.) *infantum* activity of compounds **1**–**4** was determined, and physicochemical properties were studied via an *in silico* approach. Finally, insight into the mechanism of lethal action of the most potent compound **2** was obtained using spectrofluorimetric assays, flow cytometry, and transmission electron microscopy, and its immunomodulatory potential in macrophages was also investigated.

## Results

### *In vitro* anti-*L*. (*L*.) *infantum* activity and mammalian toxicity

The activity of the four semi-synthetic derivatives against intracellular amastigotes of *L*. (*L*.) *infantum* was evaluated by light microscopy counting. The studied compounds were effective with 50% inhibitory concentration (IC_50_) values between 6.1 and 35.9 μM (Table 1). In the promastigote assays, the activity was evaluated using the MTT method and the results showed that all four compounds killed 100% of the parasites at the highest concentration. The IC_50_ values for the promastigote forms ranged from 29 to 105.4 μM. Miltefosine was used as standard and presented IC_50_ values in intracellular amastigotes and promastigotes of 6.5 and 5.1 μM, respectively. Compounds **1** and **3** showed cytotoxicity at the tested concentrations, with 50% cytotoxic concentration (CC_50_) values of 75.0 and 57.7 µM, respectively. Considering the relationship between activity against intracellular amastigotes, and cytotoxicity in NCTC cells, it is possible to calculate the selectivity index (SI) of the compounds, which ranged from 2.1 to >32.8. Compound **2** was found to be the most potent and selective compound, and was therefore selected for lethal action studies. A second IC_50_ value for compound **2** against promastigotes was evaluated (190 µM) after 2 h of incubation, and used for following mechanism of action assays.

**Table 1:** Antileishmanial activity and mammalian cytotoxicity of the semi-synthetic compounds **1**–**4**.

Compound	IC_50_ (µM) ± SD		CC_50_ (µM) ± SD NCTC	SI
	Promastigote	Amastigote		
1	93.3 ± 8.4	35.9 ± 6.6	75.0 ± 13.8	2.1
2	105.4 ± 9.4	6.1 ± 1.2	>200	>32.8
3	29.6 ± 2.3	21.7 ± 1.9	57.7 ± 1.1	2.7
4	29.0 ± 0.2	34.5 ± 11.4	>200	>5.8
Miltefosine	5.1 ± 0.6	6.5 ± 3.0	119.7 ± 4.2	18.4

IC_50_: 50% Inhibitory Concentration; CC_50_: 50% Cytotoxic Concentration; SI: Selectivity Index, given by the ratio between CC_50_ in NCTC cells and IC_50_ in intracellular amastigotes; SD: Standard Deviation.

### *In silico* analysis

Semisynthetic analogs **1**–**4** were evaluated *in silico* using two web-based platforms, FAF-Drugs4 and ADMETlab, to identify their potential for pharmacokinetic or toxicologic risks or liabilities (Table 2). All four semi-synthetic derivatives are predicted to have good oral bioavailability based on twelve physicochemical descriptors for oral drugs. None of the compounds contain structural similarities to pan-assay interference compounds (PAINs). The compounds are predicted to be non-mutagenic by the AMES test and non-inducers of phospholipidosis. Toxicity alerts associated with high lipophilicity were predicted and include: hERG, DILI, and human hepatoxocity. Structural modifications that reduce lipophilicity can eliminate or reduce these toxicities. As such, these toxicity alerts are not major at this stage. Compounds **1** and **2** were predicted to inhibit cytochrome P450 enzymes. Application of the Lilly Med Chem rules identified the phenolic ester of compound **1** as a risk. It is predicted to metabolize *in vivo* to yield a phenol. Overall, the *in silico* predictions indicate that compounds **1**–**4** represent a promising, orally bioavailable scaffold with no major risks predicted.

**Table 2:** *In silico* prediction of druglike properties.

Properties	1	2	3	4
MW	382.5	420.5	330.4	340.4
LogP*	5.76	7.29	5.72	5.38
LogP	5.27	6.98	5.11	5.57
TPSA**	54	36.9	47.9	36.9
Solubility**	low	low	low	low
Permeability (Caco-2)	moderate	moderate	moderate	moderate
Pgp inhibitor	yes	yes	yes	yes
Pgp substrate	yes	yes	yes	yes
HIA (>/=30%)	yes	yes	yes	yes
Oral bioavailability	≥30% F	≥20% F	≥30%	≥30%
Protein binding	moderate	moderate	moderate	moderate
Distribution (V_D_)	low	good	good	good
CYP3A4 inhibitor	yes	yes	no	no
CYP2C9 inhibitor	no	yes	no	no
CYP3A4 substrate	yes	yes	yes	yes
CYP2C9 substrate	yes	yes	yes	yes
Clearance	low	low	low	low
hERG	yes	yes	yes	yes
Hepatotoxicity	yes	no	yes	yes
AMES	no	no	no	no
DILI	yes	yes	yes	yes
Phospholipidosis*	no	no	no	no
Lilly Med Chem Rules*	phenolic ester	pass	pass	pass
PAINs*	no	no	no	no

ADMETlab was used to predict all values except: *Predicted using FAF-drugs4 and **Predicted using both ADMETlab and FAF-drugs4.

### Hemolytic activity

Hemolytic activity was evaluated by a colorimetric assay using BALB/c mice erythrocytes. Even after 2 h of treatment with compound **2**, no hemolytic activity could be detected in the range between 1.6 to 200 μM (data not shown).

### Mechanism of action studies

#### Plasma membrane integrity

**Figure 2:**
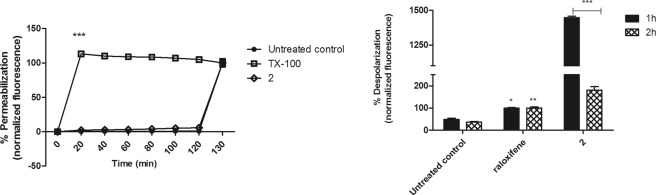
Evaluation of plasma membrane permeabilization and potential in *L*. (*L*.) *infantum* promastigotes treated with compound **2** (190 μM). (**A**) The entrance of SYTOX Green dye was monitored spectrofluorimetrically (excitation 485 nm and emission 520 nm) every 20 min. Untreated promastigotes and treated with TX-100 (0.5%) were used to achieve minimal and maximal permeabilization, respectively. Fluorescence is reported as percentage relative to time 0 min (0%) and 120 min (100%). At 120 min, the addition of 0.5% TX-100 in all samples is represented. (**B**) DiSBAC2(3) dye fluorescence was measured by flow cytometry (excitation 488 nm and emission 574 nm) after 1 and 2 h of incubation. Untreated promastigotes and treated with raloxifene (60 μM) were used to achieve minimal and maximal depolarization, respectively. Fluorescence is reported as percentage relative to promastigotes treated with raloxifene (100%). A representative experiment is shown. *p < 0.05, **p < 0.01 e ***p < 0.0001.

Damages in the plasma membrane permeability of *L*. (*L*.) *infantum* promastigotes were assessed using the fluorophore Sytox Green. No changes in the fluorescence levels were observed after treatment with compound **2**, when compared to the untreated parasites (Fig. 2A). Therefore, the compound showed no influence on the plasma membrane permeability during 120 min of treatment. Triton X-100 was used to obtain the maximum permeabilization.

#### Plasma membrane electric potential $({\rm{\Delta }}{{\rm{\Psi }}}_{{\rm{p}}})$

By flow cytometry analysis using the probe DiSBAC_2_(3), changes in the Δψ_p_ of *L*. (*L*.) *infantum* promastigotes were investigated. According to membrane depolarization, dye fluorescence increases can be verified. At both treatment times (1 and 2 h), compound **2** induced a significant (p < 0.0001) depolarization of the potential when compared with untreated parasites (Fig. 2B). In addition, it was observed that the depolarization caused by compound **2** changed after 2 h, decreasing the fluorescence levels in a time-dependent manner. Raloxifene was used as a positive control and caused increased levels of fluorescence.

#### Mitochondrial membrane electric potential $({\rm{\Delta }}{{\rm{\Psi }}}_{{\rm{m}}})$

**Figure 3:**
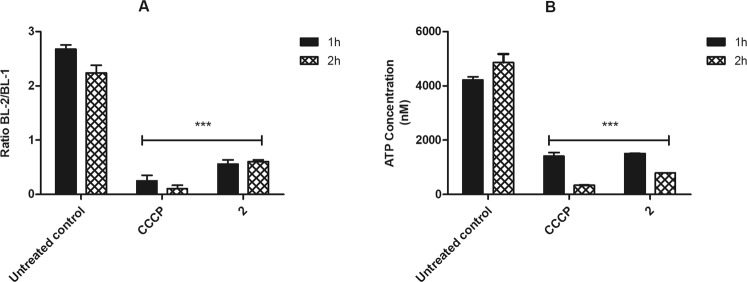
Evaluation of mitochondrial membrane potential in *L*. (*L*.) *infantum* promastigotes treated with compound **2** (190 μM). (**A**) JC-1 dye fluorescence was measured by flow cytometry (excitation 488 nm and emission 530/574 nm) after 1 and 2 h of incubation. Untreated promastigotes and treated with CCCP (100 μM) were used to achieve minimal and maximal depolarization, respectively. Fluorescence is reported as the ratio between the emission channels BL2/BL1. (**B**) Evaluation of ATP concentration. ATP was measure spectrofluorimetrically after 1 and 2 h of incubation. Untreated promastigotes and treated with CCCP (25 μM) were employ to achieve minimal and maximal depolarization, respectively. Results are expressed in nM. A representative experiment is shown. ***p < 0.0001.

The Δψ_m_ was monitored using the fluorophore JC-1, using the ratio between BL-2/BL-1 channels in flow cytometry. According to the membrane depolarization, J-aggregates formation decreases (BL-2 fluorescence) and the monomers (BL-1 fluorescence) increases, leading to a decrease in the BL-2/BL-1 ratio. The effect of compound **2** in *L*. (*L*.) *infantum* promastigotes induced a significant (p < 0.0001) depolarization of the mitochondrial membrane potential after 1 and 2 h of incubation, when compared to untreated parasites (Fig. 3A). These results were similar to that obtained with the CCCP (positive control), a known mitochondrial uncoupler.

#### ATP levels

The ATP content in *L*. (*L*.) *infantum* promastigotes was evaluated using a bioluminescence assay. The treatment with compound **2** for 1 and 2 h resulted in a dose-dependent decrease of ATP concentration (p < 0.0001), when compared to untreated parasites (Fig. 3B). Additionally, these results present a similar ATP profile to those obtained with CCCP treatment, which was used as a positive control.

#### Reactive oxygen species (ROS)

**Figure 4:**
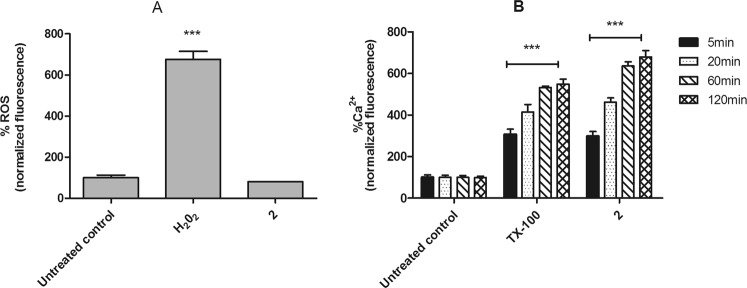
Evaluation of reactive oxygen species (ROS) and intracellular calcium levels in *L*. (*L*.) *infantum* promastigotes treated with compound **2** (190 μM). (**A**) H_2_DCFDA dye fluorescence was measured by spectrofluorimetrically (excitation 485 nm and emission 520 nm) after 2 h of incubation. Untreated promastigotes and treated with H_2_O_2_ (400 μM) were used to achieve minimal and maximal depolarization, respectively. (**B**) Fura-2 AM dye fluorescence was measured spectrofluorimetrically (excitation 360 nm and emission 500 nm) after 5, 20, 60 and 120 min of incubation. Untreated promastigotes and treated with TX-100 (0.5%) were used to achieve minimal and maximal depolarization, respectively. Fluorescence is reported as percentage relative to untreated promastigotes (100%). A representative experiment is shown. ***p < 0.0001.

The levels of ROS were determined using the fluorophore H_2_DCFDA. The *L*. (*L*.) *infantum* promastigotes treated with the compound **2**, showed similar ROS levels to those untreated parasites. H_2_O_2_ was used to obtain the maximum levels of reactive oxygen species (Fig. 4A).

#### Intracellular calcium (Ca^2+^)

The cytosolic Ca^2+^ levels were investigated in *L*. (*L*.) *infantum* promastigotes, by the changes in the florescence intensity of Fura-2 AM dye. The incubation with compound **2** induced a fast up-regulation of calcium levels, in a time-dependent manner. After five minutes of treatment, Ca^2+^ levels were significantly (p < 0.0001) higher than those obtained in the untreated parasites. Triton X-100 was used as a positive control (Fig. 4B).

#### Cell cycle analysis

**Figure 5:**
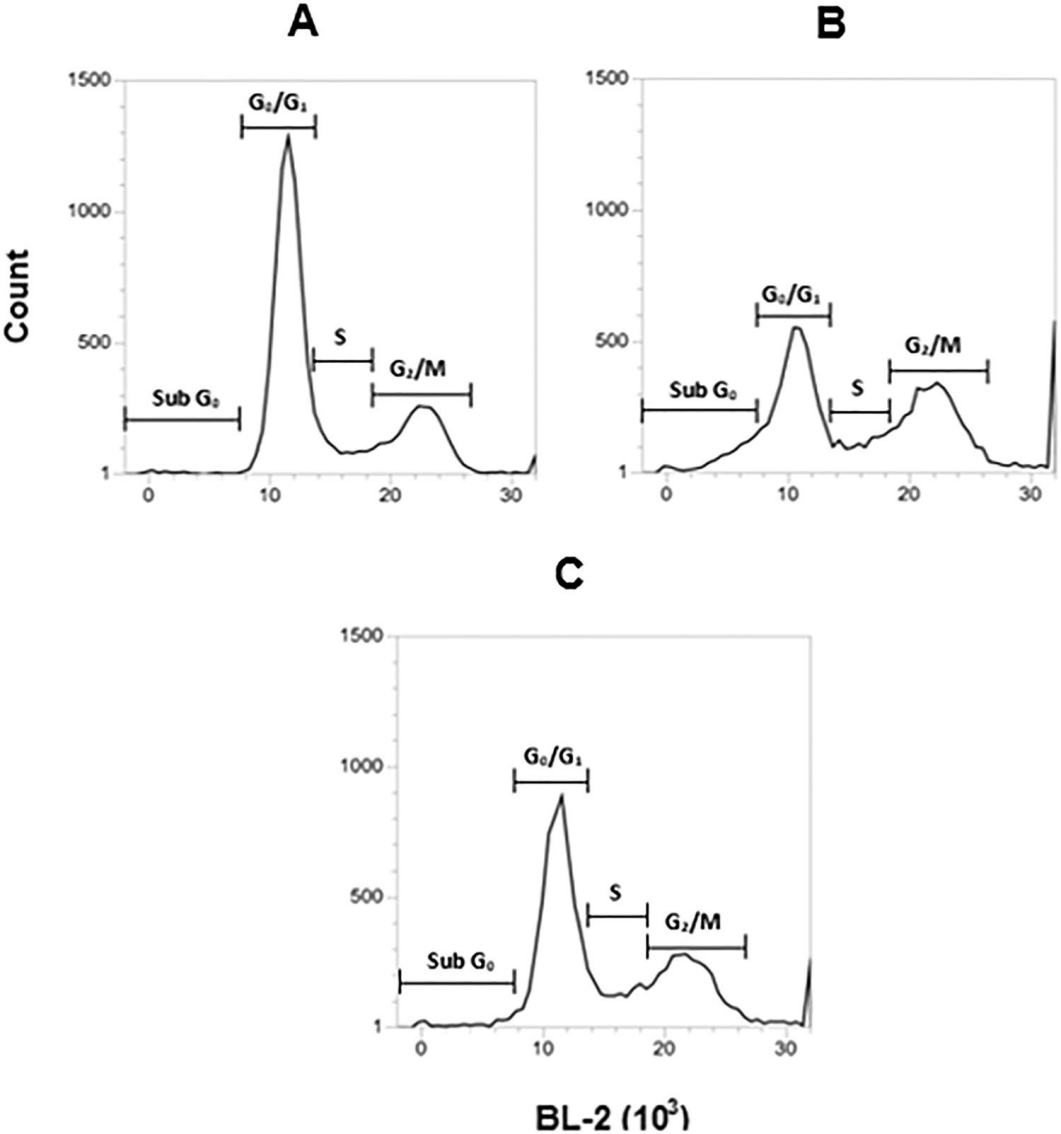
Evaluation of cell cycle progression in *L*. (*L*.) *infantum* promastigotes treated with compound **2** (190 μM) by flow cytometry (488 nm excitation and 574 nm emission). Propidium iodide fluorescence was arranged in histograms. Untreated promastigotes and treated with miltefosine (25 μM) were employing to achieve minimal and maximal depolarization, respectively. A representative experiment is shown. (**A**) Untreated control, (**B**) miltefosine, and (**C**) compound **2**. Sub G_0_: represent cells with fragmented DNA, cell death; G_0_/G_1_: diploid cells (2N); S: DNA replication; G_2_/M: cells with duplicate DNA content.

Cell cycle progression was evaluated in *L*. (*L*.) *infantum* promastigotes, using the fluorophore propidium iodide. By flow cytometry analysis, it was possible to observe an increase of BL-2 channel fluorescence, corresponding to an increased DNA content. According to the obtained results, treatment with compound **2** for 24 h resulted in significant changes (p < 0.05) in all phases of the cell cycle, when compared to untreated parasites (Fig. 5). Treatment with compound **2** clearly induced the decrease of G_0_/G_1_ cells percentage and increase of Sub G_0_, S and G_2_/M phases, a similar effect to that observed for the positive control, miltefosine (Table 3).

**Table 3:** Cell cycle analysis in *L*. (*L*.) *infantum* promastigotes treated with compound **2**.

Groups	Proportion of promastigotes in different stages of cell cycle (% ± SD)			
	Sub G_0_	G_0_/G_1_	S	M/G_2_
Untreated control	1.3 ± 0.1	67.5 ± 1.9	9.2 ± 0.3	20.9 ± 2.1
**2**	3.0 ± 0.2^**^	52.3 ± 1.1^**^	13.7 ± 0.7^**^	26.4 ± 0.1^*^
Miltefosine	10.1 ± 0.2^***^	36.4 ± 1.3^***^	12.3 ± 0.1^*^	31.4 ± 0.5^**^

Sub G0: fragmented DNA, cell death; G0/G1: diploid cells (2N); S: DNA replication; G2/M: cells with duplicated DNA content; SD: Standard deviation. *p < 0.05, **p < 0.01 and ***p < 0.001.

#### Ultrastructural studies

**Figure 6:**
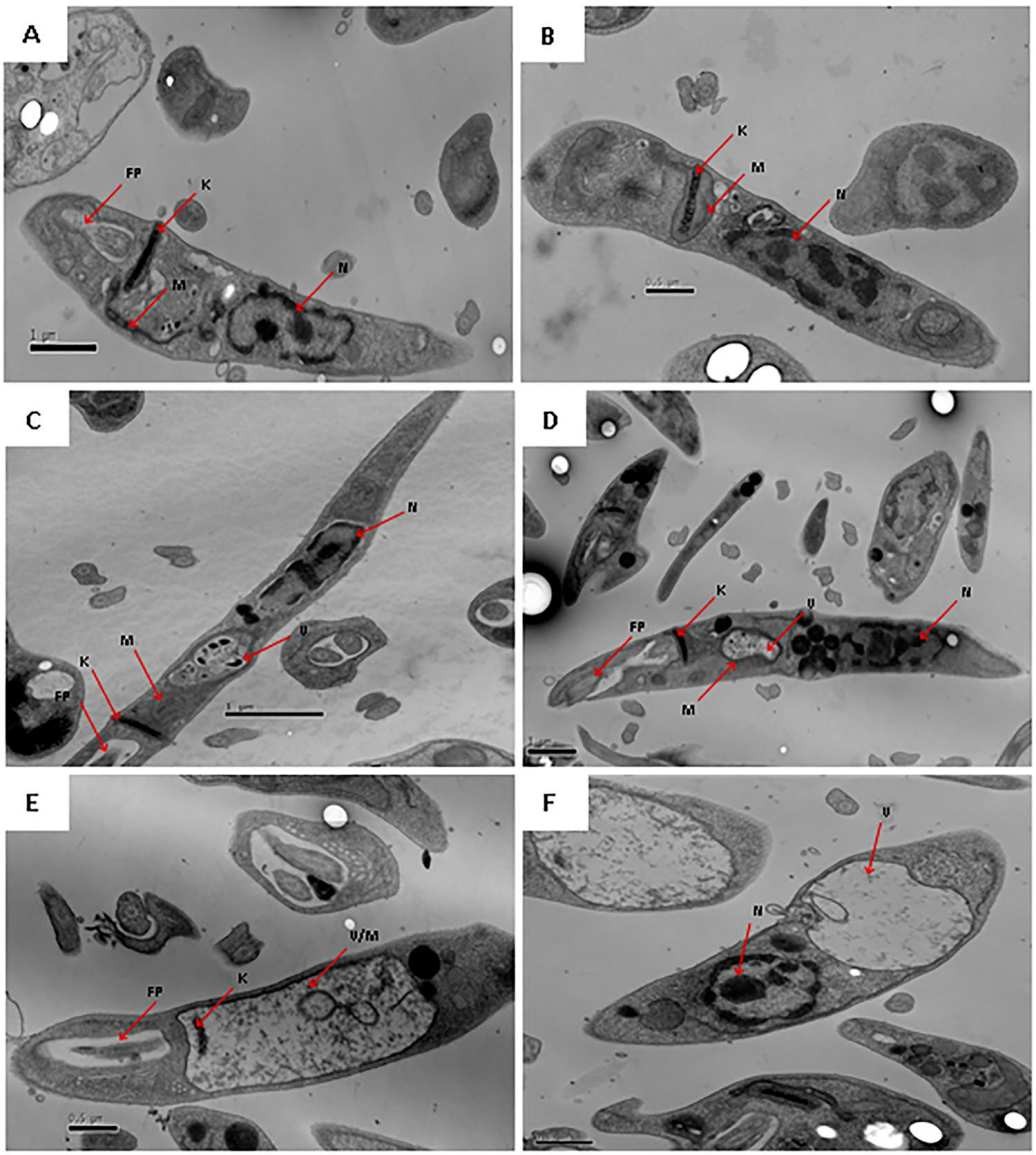
Evaluation of ultrastructural changes in *L*. (*L*.) *infantum* promastigotes treated with compound **2** (190 μM), by transmission electron microscopy. Representative images are shown. (**A**) Untreated control, (**B**) 30 min, (**C**) 1 h, (**D**) 2 h, (**E**) 4 h and (**F**) 6 h of incubation with compound **2**. K: kinetoplast; N: nucleus; M: mitochondria; V: vacuole and FP: flagellar pocket.

Using transmission electron microscopy, ultrastructural alterations of *L*. (*L*.) *infantum* promastigotes were investigated. Untreated cells demonstrated a normal morphology of cytoplasmic organelles and plasma membrane (Fig. 6A). At the initial time of incubation (30 min) with compound **2**, the mitochondria begins to swell (Fig. 6B). During the incubation period ranging from 1 to 2 h, it was possible to observe an autophagic vacuole formation, and the presence of lipid droplets aggregated around the nucleus (Fig. 6C,D). At 4 and 6 h, there was an intense swelling of the mitochondria with severe loss of cristae and matrix and concentric membranous structures inside this organelle (Fig. 6E,F). Despite significant alterations in the mitochondria, the plasma membrane, kinetoplast DNA and flagellar pocket remained preserved, as well as the nucleus.

#### Immunomodulatory studies

**Figure 7:**
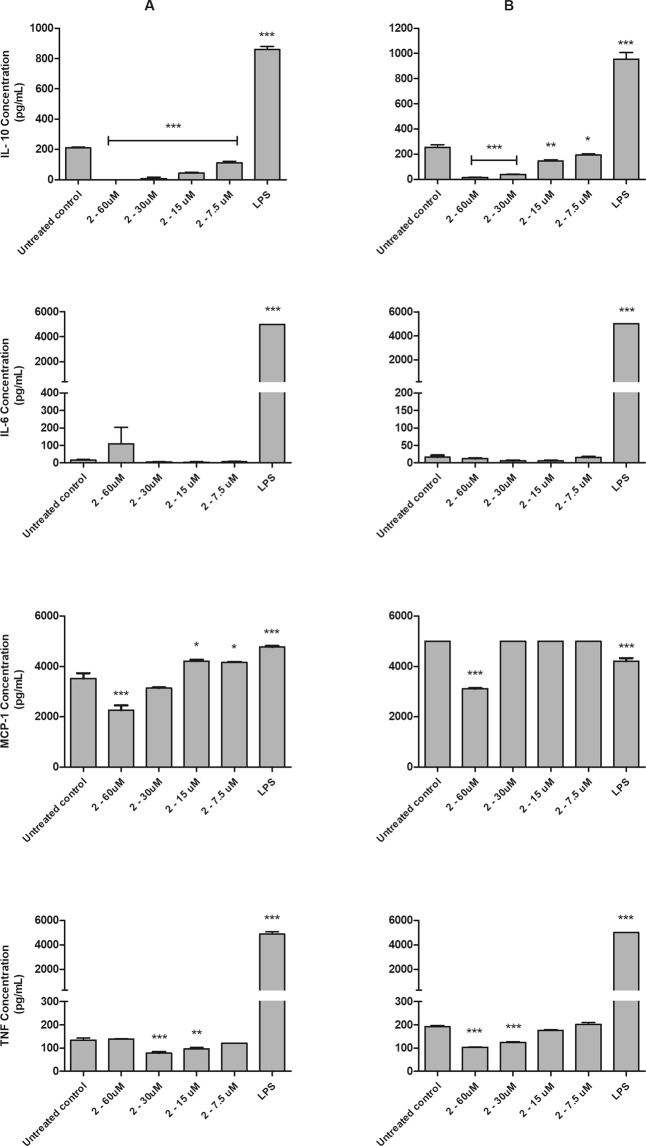
Evaluation of cytokines profile (IL-10, IL-6, MCP-1 and TNF) in bone marrow-derived macrophages treated with compound **2** (60 a 7.5 µM) and untreated. Cytokines were measure with CBA Mouse Inflammation Kit by flow cytometry after 48 h of incubation. Untreated and LPS (50 µg/mL) treated bone marrow-derived macrophages were used to achieve minimal and maximal cytokine levels, respectively. Results are expressed in pg/mL. A representative experiment is shown. (**A**) bone marrow-derived macrophages (**B**) bone marrow-derived macrophages infected with *L*. (*L*.) *infantum*. *p < 0.05, **p < 0.01 e ***p < 0.0001.

Using flow cytometry analysis, the cytokine profile of *L*. (*L*.) *infantum*-infected macrophages was determined in the presence of compound **2** (Fig. 7). Uninfected macrophages were also used for comparison. This compound induced a significant (p < 0.05) concentration-dependent decrease in the IL-10 levels of both macrophage groups, when compared to the untreated macrophages. No changes were observed in the IL-6 profile. The compound significantly (p < 0.05) reduced the TNF production of infected and uninfected macrophages at 30 and 15 μM and 60 and 30 μM, respectively. In addition, MCP-1 data demonstrated that, at an elevated concentration of 60 μM, compound **2** was able to significantly (p < 0.0001) decrease this chemokine in both macrophage groups. Conversely, at lower concentrations, the compound induced an increase of MCP-1. In the uninfected macrophages, this data was significant only at 15 and 7.5 μM, when compared to the untreated macrophages. LPS was used as positive control and increased the amount of all studied cytokines.

#### Nitric oxide (NO) levels

The NO concentrations in bone marrow-derived macrophages was determined in the culture supernatant by the colorimetric Griess assay. Both untreated and treated macrophages with compound **2** (60 to 7.5 μM) did not produced detectable levels of NO, after 48 h of incubation (data not shown). LPS was used as positive control and increased the amount of NO in the studied time.

## Discussion

A number of plant-derived secondary metabolites have been reported to show excellent antiparasitic potential against *Leishmania* parasites, including alkaloids, phenylpropanoids, saponins, flavonoids, lignoids, naphthoquinones, and iridoids^9^. In the present study, four novel derivatives of dehydrodieugenol B and methyl dehydrodieugenol B were synthesized and demonstrated activity against extracellular and intracellular forms of *L*. (*L*.) *infantum*. Compound **2** was identified as the most promising of this set, as it eliminated 100% of the amastigotes at the highest tested concentration, without affecting macrophage viability; it presented a selectivity index approximately 3 times higher than that of the dehydrodieugenol B. As compound **2** showed no toxicity to murine fibroblasts, its hemolytic activity was also evaluated in erythrocytes and the compound showed no hemolytic activity to the highest tested concentrations.

In contrast, derivatives **1** and **3** displayed significant toxicity profiles, which is likely due to the phenol motif present in these compounds. However, the maintained bioactivity of derivatives **2** and **4** shows that modification of this problematic motif is well-tolerated, providing opportunities for further development. In addition, modification of the allylic sidechains of the natural products is also possible without significant detriment to bioactivity. In light of these results, compound **2** was selected for mechanism of action studies.

*Leishmania* promastigotes were 17-fold more susceptible to compound **2** than intracellular amastigotes. Differential drug susceptibilities between promastigotes and amastigotes have been observed in literature, with^10^ or without host cell activation^11^. Besides the host cell influences, this effect could also be ascribed to the distinct metabolisms. An untargeted metabolomic study identified substantial differences between the two life stages. Compared to promastigotes, amastigotes showed decreased pools of metabolites and amino acids of the polyamine biosynthesis, alterations in the phospholipids and increased sterols. Additionally, amastigotes showed a decrease in ATP levels, kDNA mini-circles, RNA and proteins and also demonstrated lower capacity of biosynthesis, with a reduced metabolism^12^. Another comparative study of promastigotes metabolome of three *Leishmania* species, also confirmed the large differences in the extent of amino acid use and metabolism^13^. These metabolic differences can result in different drug susceptibilities between extracellular and intracellular forms of *Leishmania*, but other mechanisms related to host cells can influence the drug efficacy. In our assay, although no macrophage activation was observed, the incubation with compound **2** could have altered the intracellular transport/abundance of metabolites of the host cell, and consequently, might have affected the *Leishmania* survival in the intracellular milieu. Additionally, macrophages can also metabolize drugs, resulting in metabolites with enhanced or reduced activity/toxicity^14^. Due to this capacity, small chemical motifs can be coupled to drugs or compounds, aiming to increase the activity inside the macrophages^15^. Considering the several features that may influence the activity of compounds in the *Leishmania*-intracellular assay, additional studies will be mandatory to evaluate the action of these neolignan derivatives.

*In silico* approaches are extremely valuable for profiling new hit compounds, prioritizing experimental studies, and early risk identification. According to the selected filters, it was possible to predict some ADMET characteristics, as well as undesirable chemical groups^16^. The results obtained by the *in silico* analysis showed that the semi-synthetic derivatives present acceptable oral availability, no potential to induce phospholipidosis, are non-mutagenic, and do not resemble PAINS, corroborating previous studies with the prototype dehydrodieugenol B^6^. Risks associated with high lipophilicity were identified. The four new neolignans are predicted to exhibit hERG inhibition and drug induced liver injury (DILI). Three of the four are predicted to exhibit human hepatotoxicity. Compound **1** contains a phenolic ester, which may metabolize *in vivo* to a phenol-containing compound and, like any experimental agent, therefore requires further structural optimization. The *in silico* results enabled prioritization of follow-up studies and suggest that reducing lipophilicity, and replacement of the phenolic ester, should improve the safety of compound **1**.

Mechanism of action studies provide vital information in the drug development process and also in the search for new biochemical targets^17^. The plasma membrane regulates the transport of nutrients, ions and pH homeostasis; due to its differential chemical composition and its essential role in parasite survival, the study of plasma membrane effects becomes indispensable when investigating new hits^3^. In previous studies, plasma membrane permeabilization was verified in *Trypanosoma cruzi* parasites treated with other neolignans such as dihydrodieugenol B^8^ and 1-[(7S)-hydroxy-8-propenyl]-3-[3′-methoxy-1′-(8′-propenyl)-phenoxy]-4,5-methoxybenzene^7^. In the present work, the transmission electron microscopy data demonstrated no alteration of the *L*. (*L*.) *infantum* promastigote plasma membrane in the presence of **2**, corroborating the spectrofluorimetric study and confirming that neither pore-forming nor permeabilization activities are present.

Variations of the plasma membrane electric potential are extremely harmful to the parasite, affecting metabolite transportation and reducing the acquisition of essential nutrients^18^. The results obtained in this study demonstrate that compound **2** caused an intense depolarization of *L*. (*L*.) *infantum* promastigote plasma membrane potential, with a biological tendency for polarization as the incubation time increases. In this context, it is possible to suggest that compound **2** induced a reversible depolarization in *Leishmania* due to its penetration into the cell.

Unlike mammalian cells, trypanosomatids present single mitochondria that are essential for survival, making this organelle a target for new chemotherapeutics^19^. In previous research, neolignans eupomatenoid-5^20^ and 1-[(7*S*)-hydroxy-8-propenyl]-3-[3′-methoxy-1′-(8′-propenyl)-phenoxy]-4,5-methoxybenzene^8^ were found to induce depolarization of the mitochondrial membrane in *L*. (*L*.) *amazonensis* and *Trypanosoma cruzi* parasites, respectively. In the present study, it was possible to verify an intense depolarization induced by compound **2**. In addition, transmission electron microscopy studies have confirmed that compound **2** caused an intense swelling of mitochondria, with loss of cristae and matrix at later incubation times. Due to the early mitochondrial changes, this organelle might be a possible target of compound **2** in *Leishmania*.

Adenosine triphosphate (ATP) is a universal mediator of metabolism and signaling, being produced through the oxidative phosphorylation^21^. Depolarization of the mitochondrial membrane potential induces the collapse of the respiratory chain and lower ATP levels could generate a breakdown in the parasite metabolism, leading to cell death^22,23^. The present study also demonstrated a time dependent decrease in ATP levels in promastigotes treated with compound **2**. Mitochondria is also the largest source of reactive oxygen species (ROS)^24^; in excess, these species can cause irreversible cellular damages^21^. In the present study, promastigotes treated with compound **2** showed no alteration of ROS, suggesting that antioxidant metabolism can regulate these levels.

Calcium ions (Ca^2+^) are important in the regulation of several signaling pathways, and are essential for trypanosomatid survival^25^. The intramitochondrial Ca^2+^ concentration is responsible for several key-enzymes activation; an exacerbated increase of its concentration induces the formation of high conductance channels across the mitochondrial membranes, leading to the electrical potential dissipation^26,27^. In the present study, a time-dependent increase of calcium levels was observed in the treated parasites, suggesting that mitochondrial membrane depolarization may be ascribed to this effect.

The use of chemotherapeutic agents that target the cell division mechanism can cause serious cellular disorders, leading to the parasite death or proliferation inhibition. Promastigotes treated with compound **2** resulted in an increased number of cells in Sub G_0_ phase, indicating that the DNA content is fragmented. Apoptosis-like programmed cell death was reported in protozoans^28^, including *Leishmania* parasites treated with miltefosine^29^, but the increase in cells in Sub G_0_ after treatment with compound **2** was modest and could not be considered apoptosis. Additionally, the increased number of parasites in the S and G_2_/M phases and the decrease of G_0_/G_1_ cells suggests that compound **2** may impair the DNA replication mechanism, and consequently cause mitosis.

In addition to direct effects on the parasite, drugs can also activate host cell defenses, contributing to disease control^30^. In the present study, compound **2** demonstrated no capacity to stimulate host cells, suggesting a lethal mechanism independent of NO activation. Other neolignans as licarin A^31^ and dehydrodieugenol B^6^, also showed an anti-*Leishmania* effect without NO upregulation.

Cytokines play different roles during infection by *Leishmania* parasites^32^. The present results showed that compound **2** reduced IL-10 production in a concentration-dependent manner in *Leishmania*-infected and uninfected macrophages. However, IL-6 levels remained unchanged. Studies with dehydrodieugenol B also demonstrated a down-regulation of IL-10 levels in macrophages infected by *L*. (*L*.) *donovani*^6^. Considering that the decrease of IL-10 levels is a positive aspect for the disease control, the observed effect could contribute to an improved efficacy.

Decreased levels of MCP-1 and TNF was also observed in macrophages treated with compound **2**. In *Leishmania*-infected macrophages, treatment with MCP-1 induced pro-inflammatory cytokines and increased nitric oxide levels with reduced parasitic loads^33,34^. TNF is essential for the parasite growth control, and increased levels of this cytokine contribute to macrophage activation^35^. In the present study, the decrease of MCP-1 and TNF suggest a direct lethal effect of the compound towards the intracellular amastigotes, independent of host cell activation.

## Conclusion

In this work, four semi-synthetic neolignan derivatives were found to exhibit promising activity against the intracellular forms of *L*. (*L*.) *infantum*. Investigations into the mechanism of action of the most promising derivative (**2**) suggested an impairment of mitochondria and cell division machinery, and an antileishmanial efficacy that is independent of host cell activation. These results suggest that compound **2** may be a prototype for future optimization studies, and work towards this end is underway in our groups.

## Methods

### General experimental procedures

Silica gel (Merck, 230–400 mesh) and Sephadex LH-20 (Sigma-Aldrich) were used for column chromatography. For all extraction and chromatography procedures, analytical grade solvents (Merck) were used. IR spectra were obtained on a Shimadzu IR Prestige-21 spectrophotometer. 1H and 13C NMR spectra were recorded at 300 and 75 MHz respectively on a Bruker Ultrashield 300 Avance III spectrometer, and at 400 and 100 MHz respectively on a Bruker AVIIIHD 400 nanobay spectrometer. CDCl3 (Aldrich) was used as the solvent, with TMS as reference. HRESIMS spectra were measured on a Bruker Daltonics MicroTOF QII spectrometer.

### Plant material

Information concerning the source, identification and voucher number of the botanical material have been reported previously^36^.

### Extraction and isolation of dehydrodieugenol B and methyl dehydrodieugenol B

Similarly to the already described procedure^8^, the *n*-hexane extract from twigs of *N. leucantha* (10 g) was initially subjected to column chromatography on silica gel, eluted with an increasing proportion of EtOAc in *n*-hexane to afford 75 fractions (150 mL each), which were pooled in eight groups (A to H). Group C (1.3 g) was composed of pure methyl dihydrodieugenol B. Group E (1.5 g) was subjected to further CC on Sephadex LH-20 (h = 52 cm), eluting with MeOH and yielding 40 fractions, which were pooled in three groups (E1 to E3). Dihydrodieugenol B was obtained in pure form from group E2 (1.2 g).

Dehydrodieugenol B, IR (film) ν_max_ 3435, 2950, 2851, 1643, 1512, 1461, 1379, 1161, 976, 912, 831, 724, 590 cm^−1^; ^1^H NMR (CDCl_3_, 300 MHz) δ 6.89 (1H, d, *J* = 8.1 Hz, H-5′), 6.79 (1H, d, *J* = 2.0 Hz, H-2′), 6.70 (1H, dd, *J* = 8.1 and 2.0 Hz, H-6′), 6.49 (1H, d, *J* = 1.8 Hz, H-2), 6.40 (1H, d, *J* = 1.8 Hz, H-6), 5.84–6.01 (2H, m, H-8/H-8′), 5.00–5.13 (4H, m, H-9/H-9′), 3.89 (3H, s, 5-OCH_3_), 3.86 (3H, s, 3′-OCH_3_), 3.36 (2H, d, *J* = 6.6 Hz, H-7′), 3.24 (2H, d, *J* = 6.6 Hz, H-7); ^13^C NMR (CDCl_3_, 75 MHz) δ 150.4 (C-3′), 147.8 (C-5), 144.4 (C-3), 144.2 (C-4′), 137.4 (C-8′), 137.2 (C-8), 136.4 (C-1′), 135.2 (C-4), 131.0 (C-1), 120.8 (C-6′), 119.5 (C-5′), 116.0 (C-9′), 115.7 (C-9), 112.9 (C-2′), 111.8 (C-6), 107.3 (C-2), 56.2 (5-OCH_3_), 55.9 (3′-OCH_3_), 40.0 (C-7), 39.9 (C-7′); HRESIMS *m/z* 327.1595 [M + H]^+^ (calc. for C_20_H_23_O_4_, 327.1596).

Methyl dehydrodieugenol B, IR (film) ν_max_ 2955, 2850, 1642, 1510, 1460, 1384, 1163, 978, 915, 832, 724, 593 cm^−1^; ^1^H NMR (CDCl_3_, 300 MHz) δ 6.81 (1H, d, *J* = 8.1 Hz, H-5′), 6.69 (1H, dd *J* = 8.1 and 2.0 Hz, H-6′), 6.79 (1H, d, *J* = 2.0 Hz, H-2′), 6.48 (1H, d, *J* = 1.8 Hz, H-2), 6.27 (1H, d, *J* = 1.8 Hz, H-6), 5.83–6.03 (2H, m, H-8/H-8′), 5.00–5.13 (4H, m, H-9/H-9′), 3.87 (6H, s, 4-OCH_3_/3′-OCH_3_), 3.83 (3H, s, 5-OCH_3_), 3.37 (2H, d, *J* = 6.6 Hz, H-7′), 3.24 (2H, d, *J* = 6.6 Hz, H-7); ^13^C NMR (CDCl_3_, 75 MHz) δ 153.5 (C-3′), 150.6 (2C, C-5/C-4′), 144.1 (C-3), 138.1 (C-4), 137.4 (C-8), 137.1 (C-8′), 136.0 (C-1′), 135.5 (C-1), 120.7 (C-6′), 119.4 (C-5′), 115.9 (2C, C-9/C-9′), 113.1 (C-2′), 111.4 (C-6), 107.3 (C-2), 61.0 (4-OCH_3_), 56.1 (5-OCH_3_), 56.0 (3′-OCH_3_), 40.1 (C-7′), 40.0 (C-7); HRESIMS *m/z* 341.1753 [M + H]^+^ (calc. for C_21_H_25_O_4_, 341.1753).

### Preparation of semi-synthetic compounds

The compounds are shown in Fig. 1.

#### 1-Allyl-3-(1′-allyl-3′-methoxyphenoxy)-5-methoxyphenyl-4-propionate (**1**)

To dehydrodieugenol B (24 mg, 0.071 mmol) in CH_2_Cl_2_ (0.12 mL) at 0 °C was added Et_3_N (19 µL, 0.14 mmol, 2.0 equiv.), 4-dimethylaminopyridine (DMAP) (0.8 mg, 7.0 µmol, 0.1 equiv.), and propionyl chloride (6.2 µL, 0.071 mmol, 1.0 equiv.). The reaction mixture was warmed to room temperature, and stirred for 3 h. The mixture was then concentrated, and the resulting residue was dissolved in water (1 mL) and extracted with EtOAc (4 × 1 mL). The combined organic phases were dried (MgSO_4_), filtered and concentrated. The crude product was purified by flash chromatography (silica gel, petroleum ether/EtOAc 8:2 eluent) to afford **1** (17.5 mg, 0.046 mmol, 64%) as a pale yellow oil. IR (film) ν_max_ 2938, 1766, 1638, 1506, 1453, 1338, 1183, 1128, 914, 882, 752 cm^−1^; ^1^H NMR (CDCl_3_, 400 MHz) δ 6.85 (1H, d, *J* = 8.1 Hz, H-5′), 6.78 (1H, d, *J* = 1.8 Hz, H-2′), 6.69 (1H, dd, *J* = 8.1 and 1.8 Hz, H-6′), 6.50 (1H, d, *J* = 1.8 Hz, H-2), 6.29 (1H, d, *J* = 1.8 Hz, H-6), 5.83–6.02 (2H, m, H-8/H-8′), 5.02–5.12 (4H, m, H-9/H-9′), 3.81 (6H, s, 3′-OCH_3_/5-OCH_3_), 3.36 (2H, d, *J* = 6.7 Hz, H-7), 3.26 (2H, d, *J* = 6.7 Hz, H-7′), 2.53 (2H, q, *J* = 7.5 Hz, CH_2_CH_3_), 1.18 (3H, t, *J* = 7.5 Hz, CH_2_CH_3_); ^13^C NMR (CDCl_3_, 100 MHz) δ 172.1 (C=O), 152.4 (C-3′), 150.8 (C-5), 150.1 (C-4′), 143.7 (C-3), 138.2 (C-4), 137.3 (C-8), 136.8 (C-8′), 136.5 (C-1′), 128.7 (C-1), 120.9 (C-6′), 120.3 (C-5′), 116.2 (C-9), 115.9 (C-9′), 113.1 (C-2′), 110.9 (C-6), 106.9 (C-2), 56.2 (5-OCH_3_), 56.1 (3′-OCH_3_), 40.2 (C-7′), 40.0 (C-7), 27.1 (CH_2_CH_3_), 9.2 (CH_2_CH_3_); HRESIMS *m/z* 405.1669 [M + Na]^+^ (calc. for C_23_H_26_O_5_Na 405.1672).

#### 1-Propyl-3-(1′-propyl-3′-methoxyphenoxy)-5-methoxy-4-benzoyloxybenzene (**2**)

To a suspension of NaH (3.6 mg, 60% in mineral oil, 0.090 mmol, 1.5 equiv.) in DMF (0.3 mL) at 0 °C was added compound 3 (20 mg, 0.060 mmol). Benzyl bromide (11 µL, 0.10 mmol, 1.67 mmol) was added, and the mixture was stirred at 0 °C for 3 h. After this period, the reaction was quenched by addition of NH_4_Cl (1 mL, aq., sat.) and extracted with EtOAc (3 × 1 mL). The combined organic extracts were dried over MgSO_4_ and concentrated. The crude product was purified by flash chromatography (silica gel, petroleum ether/EtOAc 8:2 eluent) to afford **2** (19.9 mg, 0.048 mmol, 80%) as a pale yellow oil. IR (film) ν_max_ 2957, 2870, 1585, 1507, 1452, 1376, 1212, 1154, 1098, 995, 825, 731, 666 cm^−1^; ^1^H NMR (CDCl_3_, 400 MHz) δ 7.39–7.26 (5H, m, ArH), 6.77 (1H, d, *J* = 1.9 Hz, H-2′), 6.74 (1H, d, *J* = 8.1 Hz, H-5′), 6.66 (1H, dd, *J* = 8.1 and 1.9 Hz, H-6′), 6.48 (1H, d, *J* = 1.9 Hz, H-2), 6.33 (1H, d, *J* = 1.9 Hz, H-6), 5.04 (2H, s, ArCH_2_O), 3.83 (3H, s, 3′-OCH_3_), 3.80 (3H, s, H-5′), 2.56 (2H, t, *J* = 7.5 Hz, H-7′), 2.45 (2H, t, *J* = 7.5 Hz, H-7), 1.66 (2H, sext., *J* = 7.5 Hz, H-8′), 1.55 (2H, sext, *J* = 7.5 Hz, H-8), 0.96 (3H, t, *J* = 7.5 Hz, H-9′), 0.89 (3H, t, *J* = 7.5 Hz, H-9); ^13^C NMR (CDCl_3_, 100 MHz) δ 153.7 (C-3′), 150.4 (C-5), 150.1 (C-4′), 144.2 (C-3), 138.4 (C-4), 138.4 (Ar), 138.0 (C-1′), 136.7 (C-1), 128.3 (Ar), 128.0 (Ar), 127.5 (Ar), 120.6 (C-6′), 118.7 (C-5′), 112.9 (C-2′), 111.7 (C-6), 107.5 (C-2), 75.0 (OCH_2_Ar), 56.1 (5-OCH_3_), 55.9 (3′-OCH_3_), 38.1 (C-7′), 37.9 (C-7), 24.7 (C-8′), 24.5 (C-8), 13.9 (C-9′), 13.8 (C-9); HRESIMS m/z 443.2191 [M + Na]^+^ (calc. for C_27_H_32_O_4_Na 443.2193).

#### 1-Propyl-3-(1′-propyl-3′-methoxyphenoxy)-4,5-dimethoxybenzene (**3**)

To dehydrodieugenol B (100 mg, 0.30 mmol) in EtOH (5 mL) was added 10% Pd/C (10 mg, 0.090 mmol). The reaction flask was purged with hydrogen, and the reaction was stirred for 1 hour. The solution was filtered through Celite and the filtrate was concentrated to give **3** (99 mg, 0.29 mmol, 99%) as a yellow oil, which was of sufficient purity not to require further purification. IR (film) ν_max_ 3433, 2951, 2850, 1643, 1460, 1373, 1160, 911, 830, 725, 592 cm^−1^; ^1^H NMR (CDCl_3_, 400 MHz,) δ 6.87 (1H, d, *J* = 8.1 Hz, H-5′), 6.79 (1H, d, *J* = 1.9 Hz, H-2′), 6.69 (1H, dd, *J* = 8.1 and 1.9 Hz, H-6′), 6.48 (1H, d, *J* = 1.9 Hz, H-2), 6.40 (1H, d, *J* = 1.9 Hz, H-6), 5.85 (1H, s, OH), 3.89 (3H, s, 3′-OCH_3_), 3.87 (3H, s, 5-OCH_3_), 2.56 (2H, t, *J* = 7.5 Hz, H-7), 2.45 (2H, t, *J* = 7.5 Hz, H-7′), 1.65 (2H, sext, *J* = 7.5 Hz, H-8), 1.55 (2H, sext, *J* = 7.5 Hz, H-8′), 0.95 (3H, t, *J* = 7.5 Hz, H-9), 0.89 (3H, t, *J* = 7.3 Hz, H-9′); ^13^C NMR (CDCl_3_, 100 MHz) δ 150.2 (C-3′), 147.6 (C-5), 144.3 (C-3), 143.9 (C-4′), 139.1 (C-1′), 134.8 (C-4), 133.8 (C-1), 120.7 (C-6′), 119.3 (C-5′), 112.8 (C-2′), 111.7 (C-6), 107.1 (C-2), 56.2 (5-OCH_3_), 56.0 (3′-OCH_3_), 37.9 (2 C, C-7/7′), 24.7 (C-8), 24.7 (C-8′), 13.9 (C-9), 13.7 (C-9′); HRESIMS *m/z* 353.1720 [M + Na] + (calc. for C_20_H_26_O_4_Na 353.1723).

#### 1-Prop-7-enyl-3-(1′-prop-7′-enyl-3′-methoxyphenoxy)-4,5-dimethoxybenzene (**4**)

A mixture of methyl dehydrodieugenol B (50 mg, 0.146 mmol) and solid KOH (81 mg, 1.46 mmol) in ethylene glycol (0.2 mL) was heated at 150 °C (bath temperature) for 16 hours. The reaction was cooled to room temperature, then diluted with H_2_O and extracted with EtOAc (4 × 1 mL). The combined organic extracts were dried over MgSO_4_ and concentrated. The crude product was purified by flash chromatography (silica gel, petroleum ether/EtOAc 8:2 eluent) to afford **4** (49 mg, 0.144 mmol, 98%) as a pale yellow oil. IR (film) ν_max_ 2936, 1576, 1504, 1463, 1416, 1337, 1154, 1088, 960, 784, 665, 646 cm^−1^; ^1^H NMR (CDCl_3_, 400 MHz) δ 6.96 (1H, d, *J* = 1.9 Hz, H-2′), 6.83 (2H, m, H-5′/H-6′), 6.64 (1H, d, *J* = 1.9 Hz, H-2), 6.41 (1H, d, *J* = 1.9 Hz, H-6), 6.37 (1H, dd, *J* = 15.7 and 1.9 Hz, H-7), 6.14–6.24 (2H, m, H-7′/H-8′), 6.00–6.10 (1H, m, H-8), 3.89 (3H, s, 5-OCH_3_), 3.87 (3H, s, 3´-OCH_3_), 3.85 (3H, s, 4-OCH_3_), 1.88 (3H, dd, *J* = 6.6 and 1.5 Hz, H-9′), 1.81 (3H, dd, *J* = 6.5 and 1.5 Hz, H-9); ^13^C NMR (CDCl_3_, 100 MHz) δ 153.6 (C-3′), 150.7 (C-5), 150.7 (C-4′), 144.7 (C-3), 138.8 (C-4), 134.3 (C-1′), 133.6 (C-1), 130.5 (C-7), 130.4 (C-7′), 125.5 (C-8), 125.1 (C-8′), 119.6 (C-6′), 118.6 (C-5′), 109.8 (C-2′), 108.9 (C-6), 104.5 (C-2), 61.1 (4-OCH_3_), 56.1 (5-OCH_3_), 56.0 (3′-OCH_3_), 18.4 (C-9), 18.3 (C-9′); HRESIMS *m/z* 363.1563 [M + Na]^+^ (calc. for C_21_H_24_O_4_Na 363.1567).

### Animals

Male golden hamsters (*Mesocricetus auratus*, 120 g) and female BALB/c mice (20 g) were obtained from the animal breeding facility at the Adolfo Lutz Institute-SP, Brazil. The animals were maintained in sterilized cages under a controlled environment, receiving water and food *ad libitum*. All procedures performed were previously approved by the Animal Care and Use Committee from Instituto Adolfo Lutz – Secretary of Health of Sao Paulo State (Project Number CTC 21H/2015, CEUA 04/2016) in agreement with the Guide for the Care and Use of Laboratory Animals from the National Academy of Sciences. The Animal Care and Use Committee was composed by the following members: Raquel dos Anjos Fazioli (Coordinator), Alcina Maria Liserre (Vice-Coordinator), Carmen Silvia Kira, Cristina da Silva Meira Strejevich, José Eduardo de Raeffray Barbosa, Mariana Sequetin Cunha, Roberta Morozetti Blanco, Roberto Colozza Hoffmann and Rodrigo Albergaria Réssio.

### Parasites and mammalian cell maintenance

*L*. (*L*.) *infantum* (MHOM/BR/1972/LD) promastigotes were maintained in M-199 medium (Sigma-Aldrich) supplemented with 10% fetal bovine serum (FBS, Gibco), 0.25% hemin (Sigma-Aldrich), and 5% human urine at 24 °C. Amastigotes were obtained from the spleen of golden hamsters previously infected and purified by differential centrifugation^37^. Peritoneal macrophages were collected by washing the peritoneal cavity of BALB/c mice with RPMI-1640 medium (Sigma-Aldrich) supplemented with 10% FBS, and were maintained at 37 °C in a 5% CO_2_ humidified incubator. Murine fibroblasts NCTC (clone L929, ATCC) were maintained in RPMI-1640 supplemented with 10% FBS at 37 °C in a 5% CO_2_ humidified incubator. Bone marrow-derived macrophages were isolated from long bones (femurs and tibias) of BALB/c mice and maintained for approximately seven days at 37 °C in a 5% CO_2_ humidified incubator^38^.

### Evaluation of *in vitro* anti-*L*. (*L*.) *infantum* activity

#### Amastigotes

Peritoneal macrophages (1 × 10^5^ cell/well) in 16-well slide chambers (NUNC) were infected with amastigotes at a ratio of 10:1 (amastigotes/macrophage) and treated with compounds (60 to 10 µM) for 96 h. Stained slides (Giemsa) were counted using light microscopy and IC_50_ determined by the infection index^39^. Miltefosine was used as standard and untreated cells as a negative control.

#### Promastigotes

Promastigotes (1 × 10^6^ parasites/well) in 96-well plates were incubated with the four compounds (150 to 1.2 μM) for 96 h at 24 °C. The parasite viability was determined using the MTT colorimetric method^40^. Miltefosine was used as standard, with untreated cells as a negative control. A parallel promastigote activity assay was performed for 2 h with compound **2** (200 to 1.56 μM) for mechanism of action studies. After this period of incubation with compound **2**, the parasites were washed twice with M-199 medium and the parasite viability was determined using the MTT colorimetric method for 4 h incubation at 24 °C.

### Evaluation of *in vitro* mammalian toxicity

Fibroblast NCTC cells clone 929 (6 × 10^4^ cells/well) in 96-well plates were incubated with the compounds up to 200 μM in a 5% CO_2_ humidified incubator at 37 °C. CC_50_ was determined by the MTT colorimetric method^40^. The selectivity index was determined using the following equation: CC_50_ against NCTC cells/IC_50_ against amastigotes

### *In silico* physical-chemical properties, ADMET and PAINS analysis

Pharmacokinetic and toxicological risks were predicted *in silico* using two web based servers FAF-Drugs4^41^ and ADMTETlab^42^, each server is a suite of predictive models. The FAF-Drugs4 suite includes models for the prediction of physiochemical properties, solubility, oral bioavailability, drug likeness, phospholipidosis, PAINs compounds, and Lilly Med Chem Rules^16^. The Lilly MedChem Rules consist of 275 descriptors developed by Lilly using experimental data collected over 18 years. The rules were developed to identify compounds that may interfere with biological assays such as promiscuous, fluorescent, or unstable compounds^43^. ADMETlab predictions are based on a databank of over 288k entries from DrugBank and the literature, and include solubility (LogS), permeability (Caco-2), efflux transporter (Pgp) inhibition or substrate, human intestinal absorption (HIA), bioavailability (%F), plasma protein binding (PBP), volume of distribution (VD), cytochrome P450 isoform inhibition or substrate, elimination half-life (T_1/2_), clearance (CL), hERG inhibition, human hepatotoxicity, AMES mutagenicity, and drug induced liver injury (DILI).

### Hemolytic activity

Erythrocytes were collected from BALB/c mice, seeded at a 3% suspension in 96-well plates U-shape microplate and incubated with compound **2** (200 to 1.6 μM) in PBS 1× (Sigma-Aldrich), for 2 h at 24 °C. The hemolytic activity was determined in the cell supernatant by optical density reading at 570 nm (FilterMax F5 Multi-Mode Microplate Reader, Molecular Devices). Maximum hemolysis was obtained using ultrapure distilled water and untreated erythrocytes were used as negative control^44^.

### Mechanism of lethal action assessment

#### Determination of the plasma membrane integrity

Promastigotes (2 × 10^6^ parasites/well) were incubated in 96-well black polystyrene microplates with 1 µM of Sytox Green (Molecular Probes) in HANKS’ balanced salt solution (Sigma-Aldrich) supplemented with 10 mM D-Glucose (Sigma-Aldrich, HBSS + Glu) at 24 °C for 15 min in the dark^45^. Compound **2** (190 µM) was added and the fluorescence was measured every 20 min for up to 2 h, using a fluorimetric microplate reader (FilterMax F5 Multi-Mode, Molecular Devices) with excitation and emission wavelengths of 485 and 520 nm, respectively. Maximum permeabilization was obtained using 0.5% Triton X-100 and untreated parasites were used as negative control^46^.

#### Determination of the plasma membrane electric potential $({\rm{\Delta }}{{\rm{\Psi }}}_{{\rm{p}}})$

Promastigotes (2 × 10^6^ parasites/well) were treated with compound **2** (190 µM) for 1 and 2 h in HBSS + Glu at 24 °C. DiSBAC_2_(3) (Molecular Probes) were added (0.2 µM) and the parasites were incubated by 5 min^47^. The fluorescence was measure using Attune NxT flow cytometer (Thermo Fisher Scientific) with excitation and emission wavelengths of 488 and 574 nm (BL-2), respectively. Raloxifene (60 μM) was used as positive control and untreated parasites were used as negative control^48^. Unstained parasites were used to set background fluorescence.

#### Mitochondrial membrane electric potential $({\rm{\Delta }}{{\rm{\Psi }}}_{{\rm{m}}})$ analysis

Promastigotes (2 × 10^6^ parasites/well) were treated for 1 and 2 h with compound **2** (190 µM) in HBSS + Glu at 24 °C. JC-1 dye (Molecular Probes) was added at a final concentration of 10 μM. The parasites were incubated in the dark for 20 min and washed to eliminate the non-internalized dye. The fluorescence was measure using Attune NxT flow cytometer (Thermo Fisher Scientific) with excitation filter wavelengths of 488 nm and emission of 530 (BL-1) and 574 nm (BL-2). The mitochondrial membrane potential was determined using BL-2/BL-1 ratio^49^. Maximum depolarization was obtained in the presence of CCCP (100 μM) and untreated parasites were used as negative control. Unstained parasites were used to set background fluorescence.

#### Measurement of ATP levels

Promastigotes (2 × 10^6^ parasites/well) were treated with compound **2** (190 µM) in HBSS + Glu for 1 and 2 h at 24 °C. Untreated parasites and treated with CCCP (25 µM) were included as negative and positive controls, respectively. The promastigotes were lysed using 0.5% Triton X-100 and mixed with a standard reaction buffer (ATP Determination Kit, Molecular Probes) containing DTT (1 mM), luciferin (0.5 mM) and firefly luciferase (1.25 µg/mL)^50^. Luminescence intensity was measured using a luminometer (FilterMax F5 Multi-Mode, Molecular Devices) and the amount of ATP was calculated from an ATP standard curve.

#### Measurement of reactive oxygen species (ROS) generation

Promastigotes (2 × 10^6^ parasites/well) were seeded in 96-well black polystyrene microplates and treated with compound **2** (190 µM) for 2 h in HBSS + Glu at 24 °C. Then, H_2_DCFDA (Molecular Probes) were added (5 µM) and after 15 min of incubation, the fluorescence was measure using a fluorimetric microplate reader (FilterMax F5 Multi-Mode, Molecular Devices) with excitation and emission wavelengths of 485 and 520 nm, respectively^49^. H_2_O_2_ (400 μM) was used as positive control and untreated parasites were used as negative control.

#### Measurement of intracellular calcium levels (Ca^2+^)

Promastigotes (2 × 10^6^ parasites/well) were pretreated with 5 µM of Fura-2 AM (Molecular Probes) in PBS 1x, for 40 min at 24 °C in the dark. The parasites were washed and treated with compound **2** (190 µM). The fluorescence was measured at 5, 20, 60 and 120 min, using a fluorimetric microplate reader (FilterMax F5 Multi-Mode, Molecular Devices) with excitation and emission wavelengths of 360 and 500 nm, respectively^51^. Maximum levels of calcium were obtained using 0.5% Triton X-100 and untreated parasites were used as negative control.

#### Cell cycle analysis

Promastigotes (2 × 10^6^ parasites/well) in mid-log phase were incubated with compound **2** (190 µM) in M-199 medium for 24 h at 24 °C. Parasites were washed and fixed in 70% ice-cold ethanol overnight at −20 °C. After a further wash with PBS 1x, the promastigotes were ressuspended in propidium iodide (10 µg/mL, Molecular Probes) and RNase A (20 µg/mL, Molecular Probes) for 30 min in the dark at room temperature. The fluorescence intensity was analyses using Attune NxT flow cytometer (Thermo Fisher Scientific) with excitation filter wavelengths of 488 nm and emission of 574 nm (BL-2)^47^. Maximum change in the cell cycle was obtained in the presence of miltefosine (25 μM) and untreated parasites were used as negative control^51^. Unstained parasites were used to set background fluorescence.

#### Ultrastructural analysis by transmission electron microscopy (TEM)

Promastigotes (2 × 10^7^ parasites/well) were treated with compound **2** (300 µM) in M-199 medium for 30 min, 1, 2, 4 and 6 h at 24 °C. Then, the parasites were washed, fixed in 2.5% glutaraldehyde in 0.1 M sodium cacodylate buffer (pH 7.3), postfixed in 1% osmium tetroxide. The parasites were dehydrated with acetone series and embedded in Epon resin. Ultrathin sections were stained with uranyl acetate and lead citrate^52^. The material was analyzed under transmission electron microscopy (JEOL JEM-1011). Untreated parasites were used as negative control.

#### Cytokine level quantification

Bone marrow-derived macrophages (5 × 10^5^ cells/well) in 24-well plates were infected with amastigotes at a ratio of 10:1 (amastigotes/macrophage) and kept at 37 °C in a 5% CO_2_ humidified incubator. Cell were treated with compound **2** (60 to 7.5 µM) for 48 h and the supernatant was collected and cytokine quantification was achieved using the CBA Mouse Inflammation Kit (BD Biosciences) according to the manufacturer’s instructions. The fluorescence was measured using BD LSRFortessa flow cytometer (BD Biosciences) and the data analysis was performed using the software FCAP Array (v.3). LPS (50 μg/mL) was used as positive control and untreated parasites were used as negative control.

#### Nitric oxide evaluation

The nitric oxide (NO) content was quantified in the supernatants collected from bone marrow-derived macrophages treated for 48 h (compound **2**–60 to 7.5 µM). The samples were analyzed by the Griess method using a microplate reader at 570 nm (FilterMax F5 Multi-Mode- Molecular Devices)^53^. The amount of NO was obtained from a standard curve prepared with NaNO_2._ Maximum nitric oxide production was obtained in the presence of LPS (25 μg/mL) and untreated parasites were used as negative control^54^.

### Statistical analysis

The determination of the CC_50_ and IC_50_ values was obtained from sigmoid dose-response curves. The statistical significance (p value) between the samples was evaluated through the One-way ANOVA method using the Tukey’s Multiple Comparison test. All analyzes were performed using Graph Pad Prism 5.0 software. The samples were tested in duplicate and the assays were repeated at least twice.
